# Trends and Demographics of Liver Fibrosis and Cirrhosis‐Related Mortality Among Adults Living in the United States From 1999 to 2020: A CDC Wonder Analysis

**DOI:** 10.1002/jgh3.70247

**Published:** 2025-09-26

**Authors:** Muhammad Shahzad, Syeda Sundus Shah Bokhari, Fnu Rabia, Amna Zaman Khan, Muhammad Abdullah Ali, Ali Hashim, Farah Shahzad, Maryam Tariq, Zarhaish Barkat‐Ullah, Malaika Rasheed, Muhammad Uzair Khan Niazi, Ali Hassan, Asfand Yar Khan, Taha Mazhar Awan, Saad Ahmed Waqas, Raheel Ahmed

**Affiliations:** ^1^ Foundation University Medical College Islamabad Pakistan; ^2^ Ayub Medical College Abbottabad Pakistan; ^3^ Allama Iqbal Medical College Lahore Pakistan; ^4^ Al‐Aleem Medical College, University of Health Sciences Lahore Pakistan; ^5^ Khyber Medical College Peshawar Pakistan; ^6^ Aziz Fatimah Medical and Dental College Faisalabad Pakistan; ^7^ Fatima Memorial Hospital College of Medicine and Dentistry Lahore Pakistan; ^8^ Department of Anatomy Foundation University Medical College Islamabad Pakistan; ^9^ Fauji Foundation Hospital Rawalpindi Pakistan; ^10^ Pak International Medical College Peshawar Pakistan; ^11^ Dow University of Health Sciences Karachi Pakistan; ^12^ National Heart and Lung Institute Imperial College London London England

## Abstract

**Introduction:**

Liver cirrhosis, the fifth leading cause of adult mortality, involves progressive, irreversible liver fibrosis and loss of function. Its rising prevalence necessitates studying trends, identifying high‐risk groups, and enhancing preventive strategies. This study aims to assess temporal trends and demographic disparities in liver fibrosis and cirrhosis‐related mortality in the United States from 1999 to 2020.

**Methods:**

Death certificates from the CDC WONDER(Centers for Disease Control and Prevention Wide‐Ranging Online Data for Epidemiologic Research) database for 1999–2020 were analyzed for liver fibrosis and cirrhosis‐associated mortality in adults > 25 years. AAMRs per 100 000 were stratified by year, sex, race/ethnicity, and region. Joinpoint Regression (v5.3.0.0) calculated annual percent change (APC) and average APC (AAPC), identifying significant trends (*p* < 0.05, two‐tailed *t* test).

**Results:**

From 1999 to 2020, 787 375 liver cirrhosis‐related deaths occurred in adults > 25. AAMR increased from 16.61 (1999) to 18.93 (2020). Men had a higher AAMR (21.51; 95% CI: 21.44 to 21.57) than women (11.73; 95% CI: 11.68 to 11.77). AAMRs were highest in Non‐Hispanic (NH) American Indian (26.42; 95% CI: 25.91 to 26.93) followed by Hispanics (24.93; 95% CI: 24.77 to 25.09), NH White (16.71; 95% CI: 16.67 to 16.75), NH Black (15.13; 95% CI: 15.02 to 15.24), and NH Asian/Pacific Islander (9.29; 95% CI: 9.15 to 9.42). By region, the South had the highest AAMR (18.87; 95% CI: 18.8 to 18.93), followed by the West (15.75; 95% CI: 15.67 to 15.82), Midwest (14.55; 95% CI: 14.47 to 14.62), and Northeast (14.17; 95% CI: 14.1 to 14.25). Micropolitan (Nonmetro) areas had the highest AAMR (17.62; 95% CI: 17.49 to 17.74), while Large Fringe Metro Areas had the lowest AAMR (14.2; 95% CI: 14.13 to 14.27). Texas reported the highest AAMR (25.7); Nebraska reported the lowest (9.4).

**Discussion:**

Liver cirrhosis‐related mortality has risen since 1999, especially among Hispanic adults, men, and those in Southern or nonmetropolitan regions. Targeted prevention is needed to reduce mortality in these high‐risk groups.

**Abbreviations**
AAMR: age‐adjusted mortality rate
APC: annual percent change
CDC WONDER: Centers for Disease Control and Prevention Wide‐Ranging Online Data for Epidemiologic Research
ICD: International Classification of Diseases
NFALD: nonalcoholic fatty liver disease
NH: non‐Hispanic
STROBE: Strengthening the Reporting of Observational Studies in Epidemiology

## 1 Introduction

Liver fibrosis is defined as the progressive accumulation of extracellular matrix proteins, including collagen, as a response to chronic liver injury. Over time, persistent fibrosis may advance to cirrhosis, the terminal stage of fibrosis, which is characterized by extensive scarring, nodule formation, and architectural distortion of the liver parenchyma, ultimately leading to hepatic dysfunction. Cirrhosis is considered an irreversible condition and is characterized by the histological formation of regenerative nodules encased by fibrous bands in response to chronic liver injury, ultimately resulting in portal hypertension and end‐stage liver disease. In venous outflow obstruction, fibrosis gradually connects adjacent central veins and portal tracts, leading to veno‐portal cirrhosis or veno‐centric cirrhosis, also known as “reversed lobulation” cirrhosis [1]. Liver cirrhosis is a prevalent chronic progressive disease with high mortality, resulting from one or more contributing factors, including chronic viral hepatitis (HBV, HCV), excessive alcohol consumption, nonalcoholic fatty liver disease (NAFLD), autoimmune liver disorders, genetic metabolic diseases, and prolonged exposure to hepatotoxic substances. Liver disease is currently the 11th leading cause of death; however, the actual mortality rate may be underestimated [2]. Liver cirrhosis ranks as the fifth leading cause of adult deaths and the primary cause of liver‐related mortality worldwide [3]. Cirrhosis currently ranks as the 10th leading cause of death in Africa, rising from 13th in 2015. It is the ninth leading cause in both Southeast Asia and Europe, and the fifth leading cause in the Eastern Mediterranean region. The prevalence of alcohol‐related cirrhosis is highest in Europe, ranging from 16% to 78% [4]. Liver disease is becoming more prevalent in the United States and globally, driven by shifting lifestyle patterns. The rate of fibrosis progression increased with age, while females generally exhibited a slower progression of liver fibrosis, except in cases of alcoholic liver disease [5]. Identifying the trends in liver fibrosis and cirrhosis‐related mortality among adults in the United States can help identify high‐risk populations, inform public health policies, improve early detection, optimize healthcare resource allocation, assess intervention effectiveness, understand lifestyle influences, and advance medical research for better patient outcomes. Therefore, the objective of this study is to assess temporal trends and demographic disparities in liver fibrosis and cirrhosis‐related mortality among US adults from 1999 to 2020 using national death certificate data.

## 2 Methods

### 2.1 Study Setting and Population

This study was conducted on secondary data of US mortality data, available on CDC WONDER (Centers for Disease Control and Prevention Wide‐Ranging Online Data for Epidemiologic Research) [6]. Data on mortality due to fibrosis and cirrhosis of the liver during 1999–2020 were retrieved using the International Classification of Diseases, 10th Revision (ICD‐10) code, K74 Fibrosis and Cirrhosis of the liver [7]. The CDC is a comprehensive database comprising data from US death certificates from all states. This paper was written as per STROBE (Strengthening the Reporting of Observational Studies in Epidemiology) guidelines for reporting [8]. This data is anonymous and is available for researchers to use.

### 2.2 Data Abstraction

Data was stratified by gender, race, age, year, and place of death, metropolitan and nonmetropolitan, and state. Data stratified by race was present under Hispanics or Non‐Hispanics (NH), and the latter were further classified as Asian or Pacific Islander, Black or African American, White, and American Indian or Alaskan Native. When stratified by urbanization, the National Center for Health Statistics Urban–Rural Classification Scheme was utilized [9].

### 2.3 Statistical Analysis

An analysis of the data was done by calculating Age‐Adjusted Mortality Rates (AAMRs) per 100 000 population (95% confidence interval). Crude death rates were also retrieved. Moreover, by using the Joinpoint regression program (Joinpoint, National Cancer Institute) annual percent change (APC) was calculated [10]. Joinpoint used log‐linear regression models to calculate temporal variations (Alpha < 0.05) and interpreted them via a two‐tailed *t* test.

## 3 Result

### 3.1 Overall Mortality Trends

**FIGURE 1:**
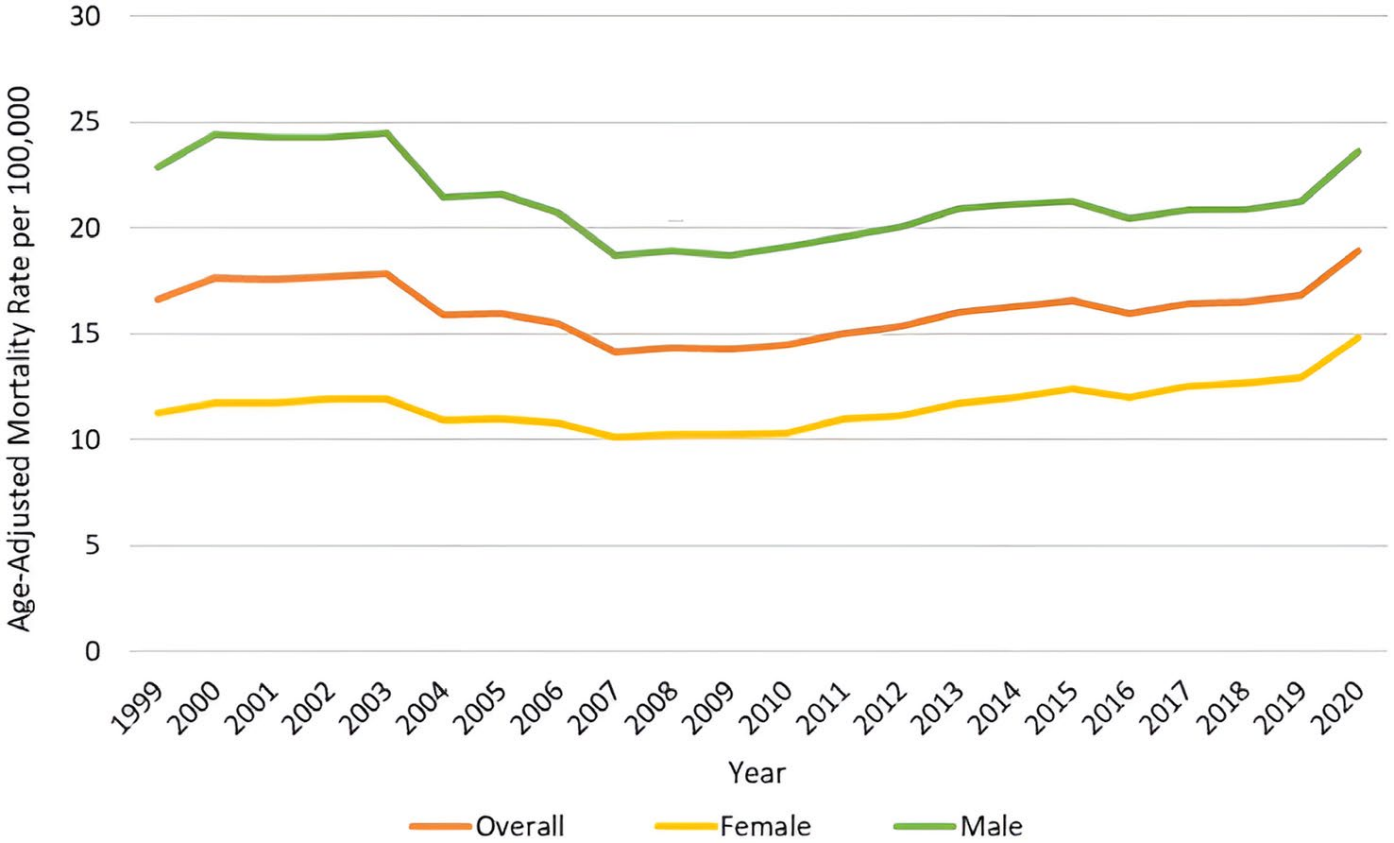
Overall and sex stratified liver cirrhosis‐related age‐adjusted mortality rates (AAMRs) per 100 000 in adults in the United States, 1999–2020. Age‐adjusted mortality rates (AAMR) related to liver cirrhosis among adults in the United States from 1999 to 2020, overall trends and stratified by sex. The x‐axis shows the calendar years (1999–2020), and the y‐axis shows the age‐adjusted mortality rate per 100 000 persons. The yellow line represents the overall AAMR per 100 000 population. The red line indicates AAMR among males, which remained consistently higher than both the overall and female rates (green line) which remained lower than the overall rate across all years.

**FIGURE 2:**
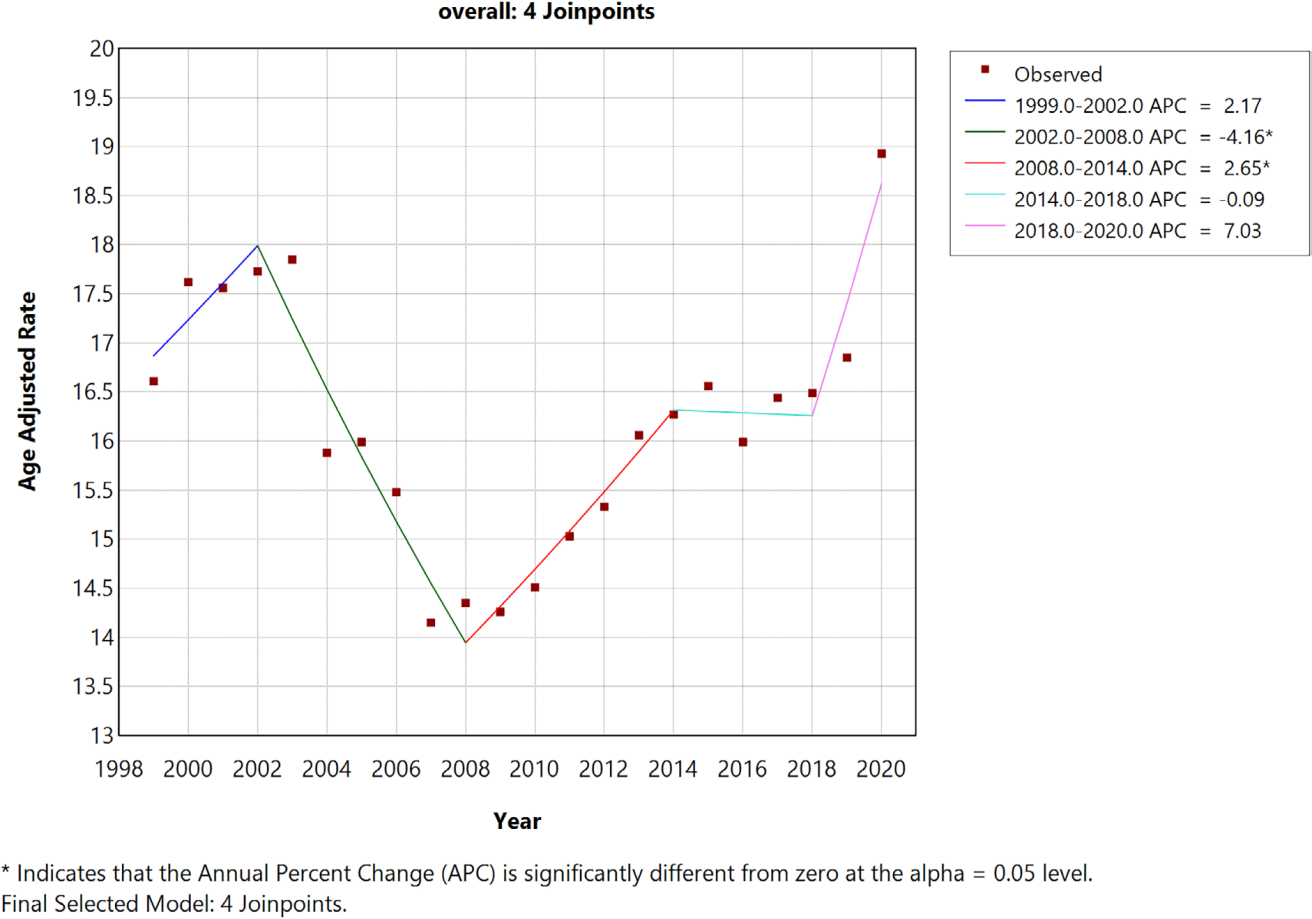
Joinpoint analysis for overall, showing Annual Percent Change (APC) (95% CI) from 1999 to 2020. Joinpoint regression analysis of age‐adjusted mortality rates (AAMRs) related to liver cirrhosis in the United States from 1999 to 2020. The x‐axis represents calendar years (1999–2020), while the y‐axis shows age‐adjusted mortality rates per 100 000 population. Annual AAMRs are marked by red points, and solid lines illustrate the fitted trends between statistically significant joinpoints, indicating APC. A legend is included to present the corresponding APC values derived from the regression analysis. The analysis identified five distinct trend segments over the study period.

A total of 787 375 deaths were observed among adults living in the United States from 1999 to 2020. The AAMR for fibrosis and cirrhosis of liver‐related deaths was 16.61 in 1999 and 18.93 in 2020. The overall AAMR increased from 1999 to 2002 (APC: 2.16; 95% CI −2.18 to 6.70), followed by a sharp decrease till 2008 (APC: −4.15; 95% CI −5.93 to −2.34), followed by a sharp increase till 2014 (APC: 2.65; 95% CI 0.85 to 4.47), then constant till 2018 (APC: −0.08; 95% CI −3.62 to 3.57) and finally a sharp increase till 2020 (APC: 7.02; 95% CI: −0.26 to 14.85) (Figures 1 and 2, Table S1).

### 3.2 Sex‐Specific Trends

**FIGURE 3:**
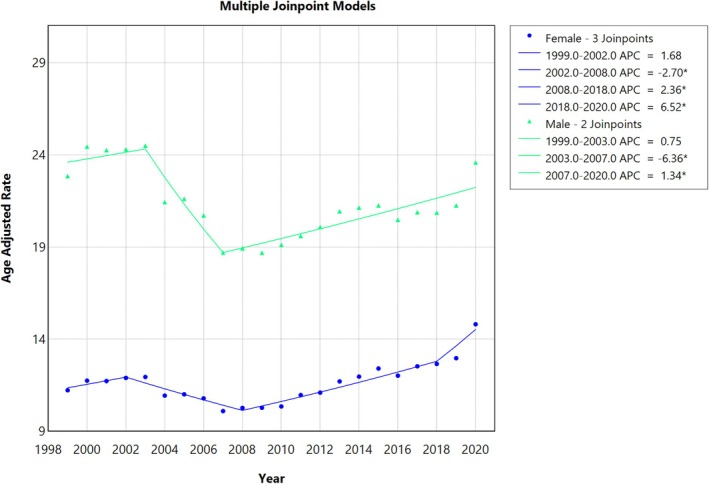
Joinpoint analysis for sex, showing Annual Percent Change (APC) (95% CI) from 1999 to 2020. Joinpoint regression analysis of liver cirrhosis‐related age‐adjusted mortality rates (AAMRs) stratified by sex in the United States from 1999 to 2020. The x‐axis represents calendar years (1999–2020), and the y‐axis displays age‐adjusted mortality rates per 100 000 population. Annual AAMRs are indicated by coloured points, while solid lines represent fitted trends between statistically significant joinpoints, revealing APC. The females (blue line) showed the lowest mortality rates than males (green line). The figure includes a legend that displays the various APC values obtained from the joinpoint regression analysis.

Men had consistently higher AAMRs than women throughout the study period (overall AAMR men: 21.51; 95% CI: 21.44 to 21.57; women: 11.73; 95% CI: 11.68 to 11.77). The AAMR for men remained at a similar rate from 1999 to 2003 (APC: 0.75; 95% CI; −2.43 to 4.04) followed by a sharp decrease from 2003 to 2007 (APC: −6.36; 95% CI −11.10 to −1.36), followed by a rise till 2020 (APC: 1.34; 95% CI 0.83 to 1.84). Likewise, the AAMR for women in 1999–2002 increased (APC: 1.67; 95% CI −2.27 to 5.78), followed by a decrease till 2008 (APC: −2.69; 95% CI −4.38 to −0.97), the AAMR then rose till 2018 (APC: 2.36; 95% CI: 1.71 to 3.01) and finally increased sharply till 2020 (APC: 6.51; 95% CI 0.32 to 13.10) (Figures 1 and 3, Table S1).

### 3.3 Race/Ethnicity‐Specific Trends

When stratified by race/ethnicity, AAMRs were highest among NH American Indian or Alaska Native, followed by Hispanic, NH White, NH Black, and NH Asian populations. (overall AAMR NH Black or African American: 15.13, 95% CI 15.02 to 15.24; Hispanic or Latino: 24.93, 95% CI 24.77–25.09; NH American Indian or Alaska Native: 26.42; 95% CI 25.91 to 26.93; NH White 16.71, 95% CI 16.67 to 16.75; NH Asian or Pacific Islander: 9.29, 95% CI 9.15 to 9.42).

#### 3.3.1 NH White Adults

The AAMR for NH White adults increased from 1999–2002 (APC: 2.44; 95% CI −3.04 to 8.24). The AAMR further decreasedin 2008 (APC: −3.72; 95% CI −6.08 to −1.29) and then finally increased till 2020 (APC of 2.28; 95% CI 1.68 to 2.88).

#### 3.3.2 NH Black Adults

The AAMR for NH Black adults remained constant with only a minor decline noted till 2003, reflecting an APC of −0.65 (95% CI: −3.92 to 2.71). It then decreased till 2008 (APC: −5.99; 95% CI −9.18 to −2.68), then increased till 2014 (APC: 2.06; 95% CI −0.25 to 4.43), further decreasing by 2018 (APC: −2.98; 95% CI −7.58 to 1.84) and finally increasing by 2020 (APC: 5.76; 95% CI −3.53 to 15.95).

#### 3.3.3 Hispanic Adults

For Hispanic adults, the AAMR showed a decline from 1999 to 2008 with an APC of −4.01 (95% CI: −5.37 to −2.62). The AAMR then increased till 2020 (APC: 0.82; 95% CI 0.08 to 1.57).

#### 3.3.4 NH Asian or Pacific Islander Adults

NH Asian or Pacific Islander Adults had the lowest mortality with the AAMR declining till 2015, corresponding to an APC of −1.81 (95% CI: −2.40 to −1.22). The AAMR then increased till 2020 (APC: 2.50; 95% CI: −0.20 to 5.28).

#### 3.3.5 American Indian or Alaska Native Adults

**FIGURE 4:**
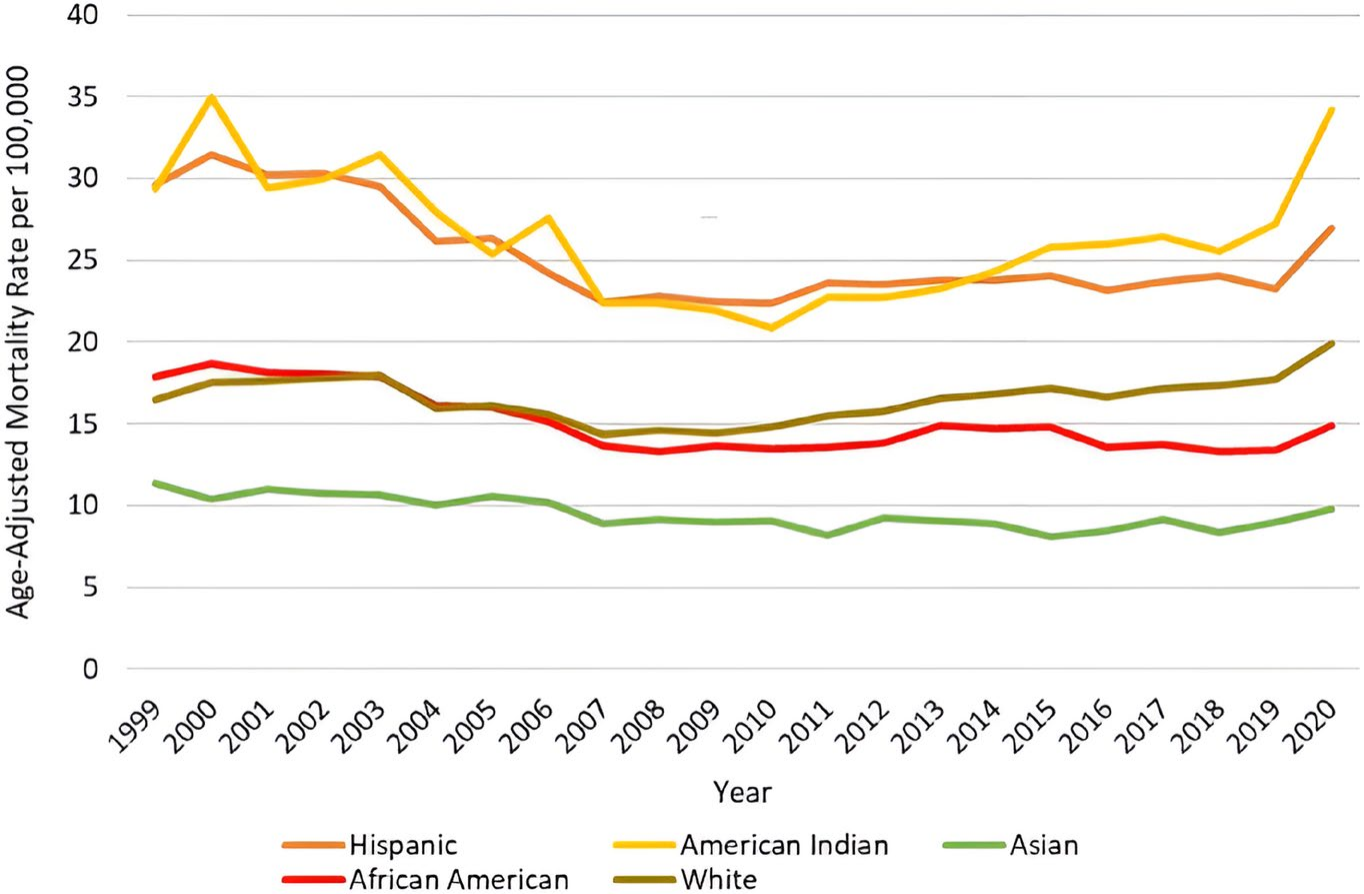
Liver cirrhosis‐related age‐adjusted mortality rates (AAMRs) per 100 000 stratified by race in adults in the United States, 1999–2020. AAMRs per 100 000 persons due to liver cirrhosis among US adults, stratified by race/ethnicity,1999–2020. The age adjusted mortality rates per 100 000 persons are noted on the y axis while the year ranges from 1999 to 2020 on the x‐axis. Non‐Hispanic American Indian adults (red line) exhibited the highest AAMR throughout most of the study period, followed by Hispanic adults (yellow line), with both groups showing early 2000s spikes—more pronounced among American Indians. A modest decline was noted from 2007 to 2014, followed by a sharp increase during 2019–2020. Non‐Hispanic White (bright green line) and Non‐Hispanic Black adults had similar trends, with higher mortality among Whites. Both groups saw minimal change until 2003; followed by a decline and then a rise during 2019–2020. Over the last decade, AAMRs were visibly lower in the Black population. Non‐Hispanic Asian adults (dark green line) consistently reported the lowest AAMRs, remaining between 5 and 10 per 100 000 persons throughout the 20‐year period.

**FIGURE 5:**
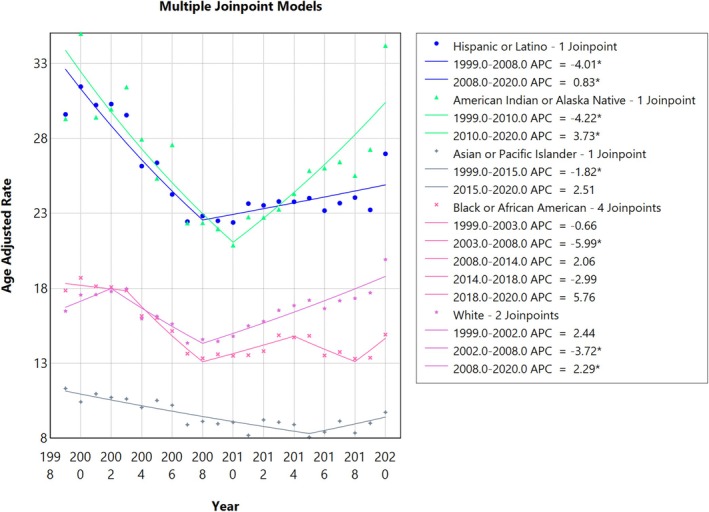
Joinpoint analysis for race/ethinicity, showing Annual Percent Change (APC) (95% CI) from 1999 to 2020. Joinpoint regression analysis of liver cirrhosis‐related age‐adjusted mortality rates (AAMRs) stratified by race/ethnicity in the United States from 1999 to 2020. The x‐axis represents calendar years (1999–2020), and the y‐axis displays age‐adjusted mortality rates per 100 000 population. Annual AAMRs are indicated by coloured points, while solid lines represent fitted trends between statistically significant joinpoints, revealing APC. The figure includes a legend that displays the various APC values obtained from the joinpoint regression analysis. Among the different ethnicities, the highest mortality rates were observed in Hispanics (blue line) and American Indians (green line), followed by the Whites (yellow lines), the Blacks (brown line), and finally the Asians (orange line), as shown by the graph.

The AAMR for American Indian or Alaska Natives declined by 2010 with an APC of −4.22 (95% CI: −5.84 to −2.57) and finally increased again up till 2020 (APC: 3.73; 95% CI: 2.13 to 5.35). (Figures 4 and 5, Table S1).

### 3.4 Urban–Rural Trends

Upon stratification by urbanization, AAMRs were highest among Micropolitan (Nonmetro) at 17.62; 95% CI: 17.49 to 17.74, followed by Medium Metro (AAMR: 17.54; 95% CI: 17.46 to 17.62), Small Metro (AAMR: 17.11; 95% CI: 16.99 to 17.23), Non‐Core (Nonmetro) with AAMR of 17.03; 95% CI: 16.89 to 17.17, Large Central Metro (AAMR: 16.55; 95% CI: 16.48 to 16.62), with Large Fringe Metro areas having the lowest AAMR (AAMR: 14.2; 95% CI: 14.13 to 14.27).

#### 3.4.1 Large Central Metro

From 1999 to 2002, the AAMR in large central metro areas slightly increased (APC: 0.40; 95% CI −3.35 to 4.30). It then decreased by 2008 (APC: −5.27; 95% CI −6.94 to −3.57). The AAMR then increased till 2014 (APC: 1.89; 95% CI: 0.08 to 3.74), followed by another decline till 2018 (APC: −1.81; 95% CI: −5.36 to 1.86) to finally increase by 2020 (APC: 6.19; 95% CI −1.09 to 14.01).

#### 3.4.2 Small Metro Areas

The AAMR increased from 1999 to 2003 (APC: 1.86; 95% CI −1.66 to 5.52). It then showed a decreasing trend from 2003 to 2008 (APC: −3.42; 95% CI −6.68 to −0.06). The AAMR increased by 2020 (APC: 2.54; 95% CI 1.96 to 3.13).

#### 3.4.3 Large Fringe Metro

There was a sharp increase in the AAMR for Large Fringe Metro from 1999 to 2002 (APC: 2.39; 95% CI: −2.10 to 7.10), followed by a sharp decrease till 2008 (APC: −4.02; 95% CI −5.89 to −2.12), to a slow increase till 2018 (APC: 1.03; 95% CI 0.26 to 1.80), and finally a sharp increase till 2020 (APC: 5.50; 95% CI: –2.05 to 13.64).

#### 3.4.4 Medium Metro

The AAMR increased from 1999 to 2002 (APC: 3.22; 95% CI −2.97 to 9.82). This was followed by a decrease till 2008 (APC: −3.68; 95% CI −6.29 to −1.00) and finally an increase by 2020 (APC: 2.43; 95% CI 1.77 to 3.09).

#### 3.4.5 Noncore (Nonmetro)

The AAMR increased from 1999 to 2002 (APC: 3.85; 95% CI −3.64 to 11.94), followed by a decrease till 2008 (APC: −2.24; 95% CI: −5.47 to 1.09), finally increasing till 2020 (APC: 3.94; 95% CI 3.14 to 4.75).

#### 3.4.6 Micropolitan (Nonmetro)

**FIGURE 6:**
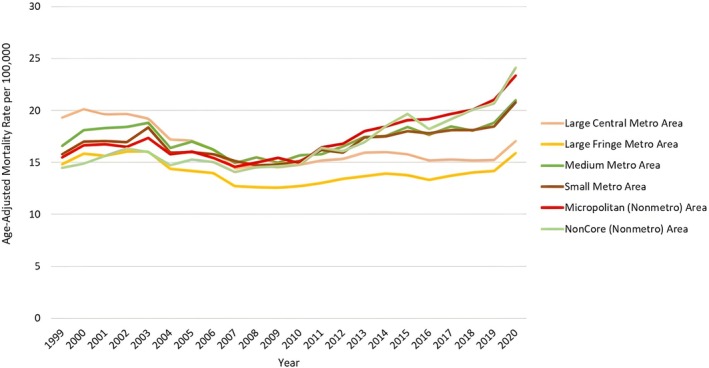
Liver cirrhosis‐related age‐adjusted mortality rates (AAMRs) per 100 000 stratified by urban–rural status in adults in the United States, 1999–2020. Figure 6 illustrates the AAMR per 100 000 persons due to liver cirrhosis among US adults, stratified by urban–rural status from 1999 to 2020. The y‐axis represents the AAMR per 100 000 persons, while the x‐axis spans the years 1999–2020. The highest overall AAMR was observed in Micropolitan (Non‐Metro) areas, represented by the red line; followed by Medium Metro areas (dark green line), Small Metro areas (light green line), Non‐Core (Non‐Metro) areas (bright green line), Large Central Metro areas (orange line), and finally, Large Fringe Metro areas, which are represented by the yellow line. Initially, Micropolitan (Non‐Metro) areas exhibited a lower AAMR, whereas Large Central Metro areas had a higher AAMR. However, this trend began to shift midway through the study period. Throughout the entire period, Large Fringe Metro areas consistently showed the lowest AAMR.

**FIGURE 7:**
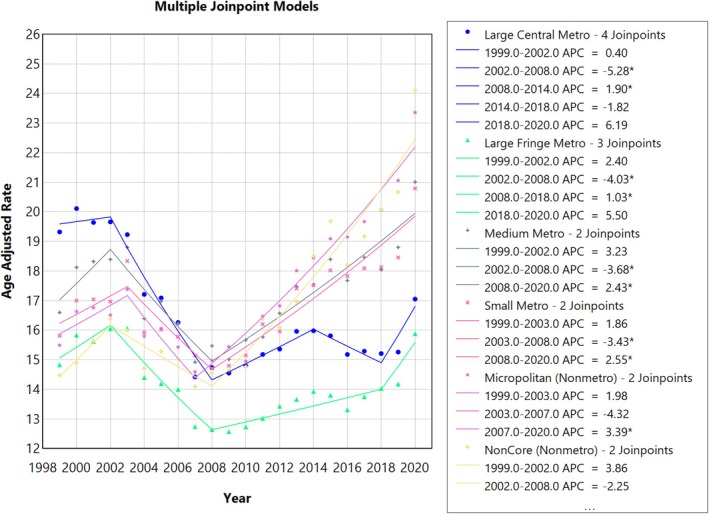
Joinpoint analysis for urbanization, showing Annual Percent Change (APC) (95% CI) from 1999 to 2020. Joinpoint regression analysis of liver cirrhosis‐related age‐adjusted mortality rates (AAMRs) stratified by urban‐rural status in the United States from 1999 to 2020. The x‐axis represents calendar years (1999–2020), and the y‐axis displays age‐adjusted mortality rates per 100 000 population. Annual AAMRs are indicated by coloured points, while solid lines represent fitted trends between statistically significant joinpoints, revealing APC. The figure includes a legend that displays the various APC values obtained from the joinpoint regression analysis. The highest mortality rates have been observed in the medium Metro areas (gray line), the Small Metro areas (pink line), the Noncore (Nonmetro) areas (yellow line) and the Micropolitan (Nonmetro)areas (purple line), with the Large Central Metro areas lying in the middle (blue line). The lowest AAMR was observed in the Large Fringe Metro areas (green line).

The AAMR increased from 1999 to 2003 (APC: 1.98; 95% CI −1.25 to 5.33), followed by a decrease till 2007 (APC: −4.32; 95% CI −8.96 to 0.55) to finally an increase by 2020 (APC: 3.38; 95% CI: 2.91 to 3.86). (Figures 6 and 7, Table S1).

### 3.5 Census/Geographical Region

**FIGURE 8:**
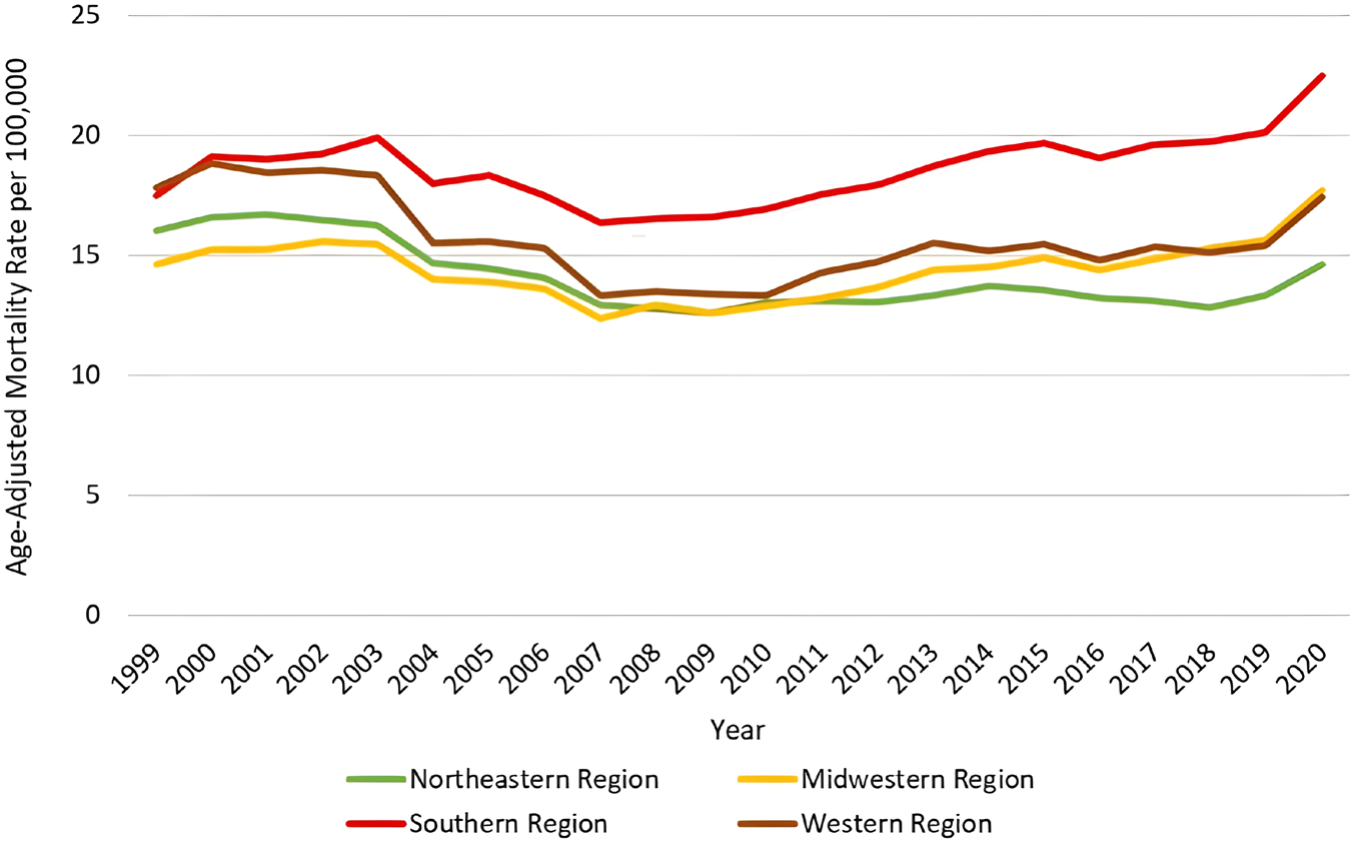
Liver cirrhosis‐related age‐adjusted mortality rates (AAMRs) per 100 000 stratified by geographical region/census from 1999 to 2020. Figure 8 presents the AAMR per 100 000 persons due to liver cirrhosis among US adults, stratified by Geographic/Census Region‐Specific Patterns from 1999 to 2020. The y‐axis displays the AAMR per 100 000 persons, while the x‐axis covers the years 1999–2020. Throughout the study period, the highest AAMR was observed in the Southern region, represented by the green line, followed by the Western region (brown line), the Midwestern region (yellow line), and finally, the Northeastern region, shown by the orange line. Initially, the Northeastern region exhibited a higher AAMR than the Midwestern region; however, around 2009–2010, this trend reversed.

**FIGURE 9:**
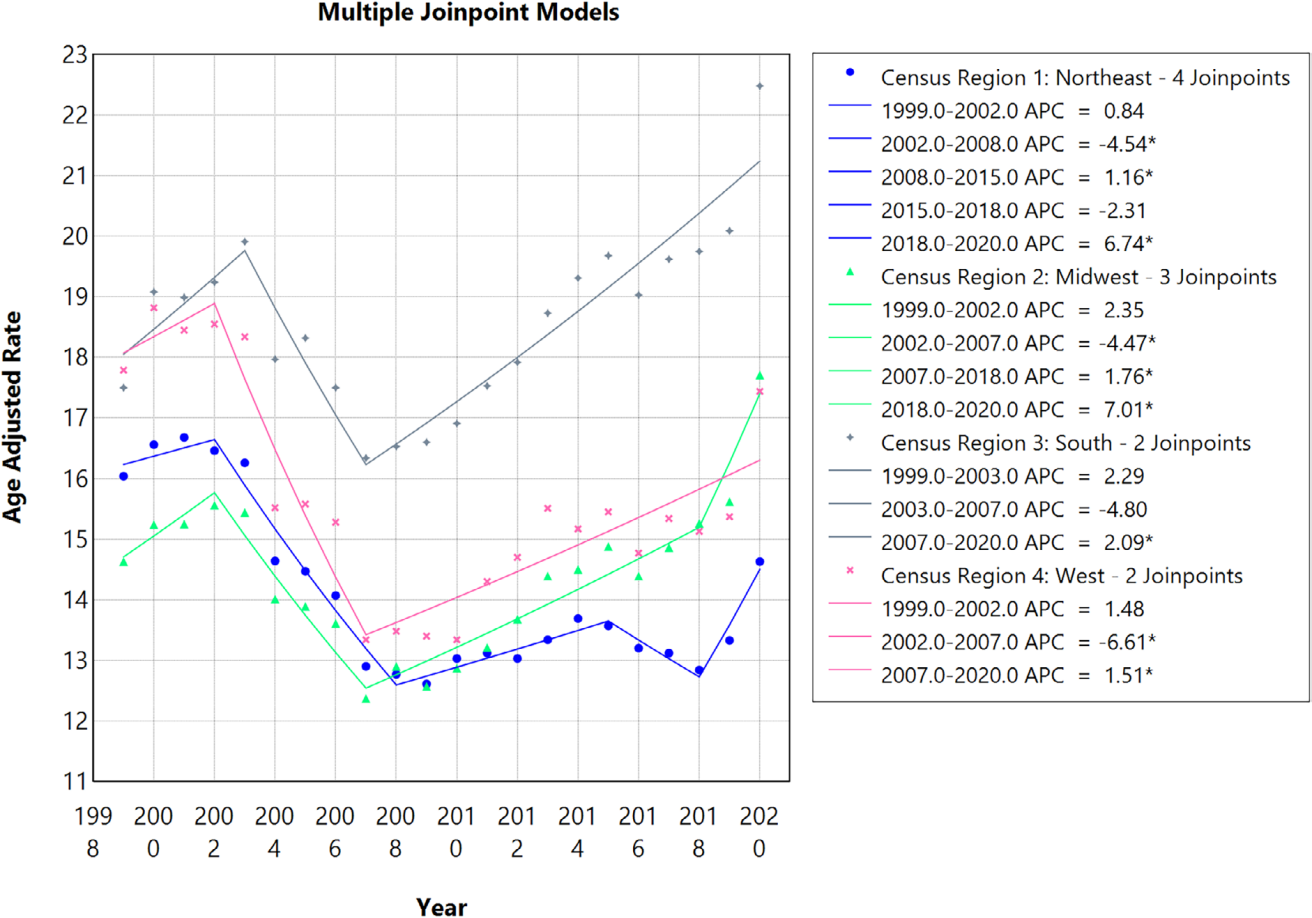
Joinpoint analysis for geographical region/census, showing Annual Percent Change (APC) (95% CI) from 1999 to 2020. Joinpoint regression analysis of liver cirrhosis‐related age‐adjusted mortality rates (AAMRs) stratified by Geographical/Census regions in the United States from 1999 to 2020. The x‐axis represents calendar years (1999–2020), and the y‐axis displays age‐adjusted mortality rates per 100 000 population. Annual AAMRs are indicated by coloured points, while solid lines represent fitted trends between statistically significant joinpoints, revealing APC. The figure includes a legend that displays the various APC values obtained from the joinpoint regression analysis. The highest mortality rates were observed in the Southern region, having two joinpoint mortality trends, followed by the Western region (pink line) exhibiting two joinpoints, and the Northeastern (blue line) with four joinpoints and Midwestern region (green line) having three joinpoints.

Throughout the study, the highest AAMR was noted in the Southern region at 18.87 (95% CI: 18.8 to 18.93), followed by the Western region at 15.75 (95% CI: 15.67 to 15.82), the Midwestern region at 14.55 (95% CI: 14.47 to 14.62), and the Northeastern region at 14.17 (95% CI: 14.1 to 14.25). In the Southern region, AAMR markedly increases from 1999 to 2003 (APC: 2.29; 95% CI: −1.14 to 5.84), followed by a sharp decrease till 2007 (APC: −4.80; 95% CI: −9.55 to 0.19) and then a sharp increase till 2020 (APC: 2.09; 95% CI: 1.59 to 2.58). In the Western region, AAMR spiked from 1999 to 2002(APC: 1.48; 95% CI −5.49 to 8.97), with a sharp decline till 2007 (APC: −6.60; 95% CI −10.66 to −2.36), followed by a sharp increase till 2020 (APC: 1.50; 95% CI: 0.80 to 2.21). In the Midwestern region, AAMR substantially increased from 1999 to 2002 (APC: 2.34; 95% CI −1.74 to 6.61), followed by a sharp decrease till 2007 (APC: −4.47; 95% CI −6.85 to −2.03), then a sharp increase till 2018 (APC: 1.76; 95% CI: 1.14 to 2.38) and then finally a further increase till 2020 (APC: 7.01; 95% CI 0.01 to 14.49). In the Northeastern region, the AAMR slightly increased from1999 to 2002 (APC: 0.83; 95% CI: −2.15 to 3.92), followed by a sharp decrease till 2008 (APC: −4.54; 95% CI: −5.87 to −3.18), then an increase till 2015 (APC: 1.15; 95% CI: 0.06 to 2.26), a minor decrease till 2018 (APC: −2.30; 95% CI: −8.05 to 3.80) and finally a sharp increase till 2020 (APC: 6.74; 95% CI: 0.64 to 13.21) (Figures 8 and 9, Table S1).

### 3.6 States

**FIGURE 10:**
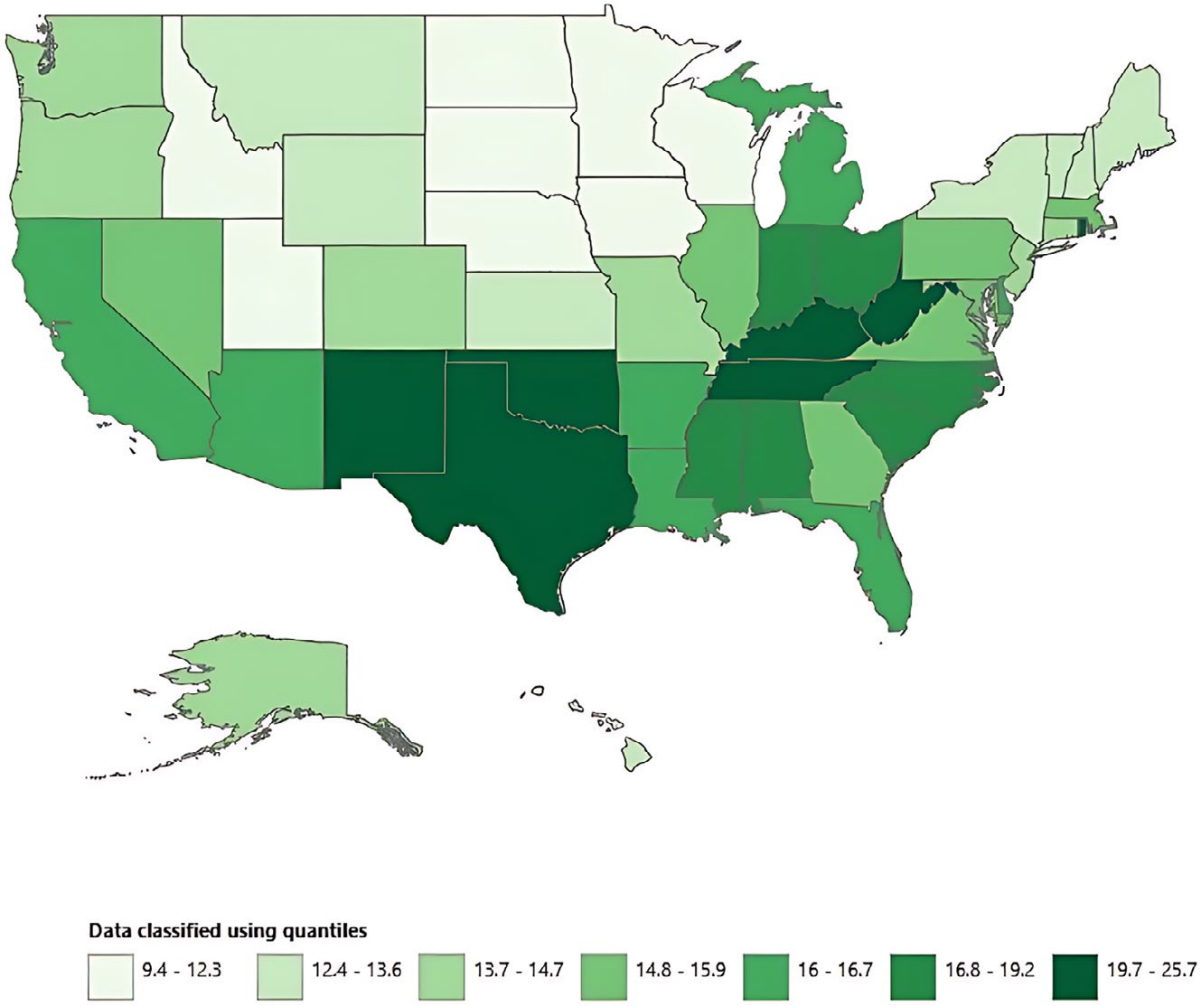
Liver cirrhosis‐related age‐adjusted mortality rates (AAMRs) per 100 000 stratified by states in adults in the United States, 1999–2020. Figure 10 displays a map of the United States sourced from the CDC WONDER database, illustrating the AAMR for each state. The data is categorized into seven quantiles, with AAMR values ranging from 9.4 to 25.8. Each state is shaded in varying tones of green corresponding to the quantile in which its AAMR falls. The highest AAMRs were recorded in West Virginia, Tennessee, Texas, Rhode Island, New Mexico, Oklahoma, Kentucky, and Alabama. In contrast, the states within the lowest quantile range (AAMR 9.4–12.3) included Idaho, Utah, Minnesota, Iowa, Nebraska, Wisconsin, North Dakota, and South Dakota.

The highest AAMR was reported by Texas (25.7), with the lowest being stated by Nebraska (9.4). Most of the states had an AAMR between 9 and 26. States having highest AAMRs were Texas (25.7), New Mexico (25.4), West Virginia (22.2), and Tennessee (20.8), which had approximately double the AAMRs compared with states with the lowest reported AAMRs, namely, Nebraska (9.4), Iowa (10.8), and North Dakota (10.8). (Figure 10, Table S2).

## 4 Discussion

**FIGURE 11:**
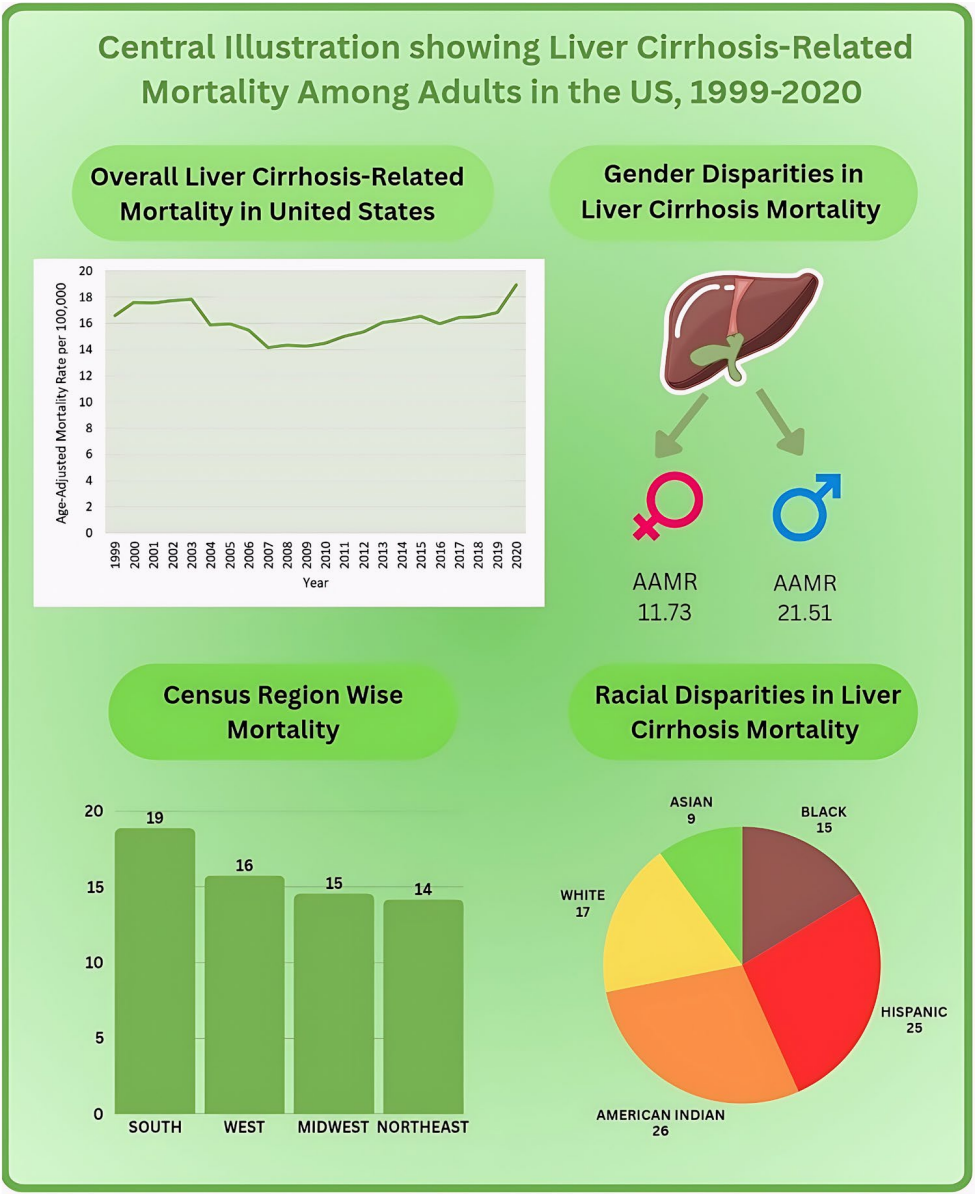
Trends and disparities in liver cirrhosis‐related mortality among adults living in the United States, 1999–2020 (age‐adjusted mortality rates [AAMRs] per 100 000 persons). 787 375 liver cirrhosis‐related deaths in adults (age ≥ 25) between 1999 and 2020. The overall AAMR has been relatively constant with sharp increase since 2018. A central illustration showing the total deaths observed in the study period, the overall AAMR observed in the form of a graph at the top, with the major trends observed stratified by different factors (race, gender, census regions, urbanization). There is also a map of the United States showing the states with the lowest AAMR (highlighted green) and the states with the highest AAMR (highlighted red). AAMR, age‐adjusted mortality rate, NH, non‐Hispanic.

In this nationwide analysis, AAMR for fibrosis and cirrhosis of liver‐related deaths increased from 1999 to 2020, showing a complex and fluctuating pattern influenced by various factors such as sex, race/ethnicity, and geographic location (Central Illustration, Figure 11). Overall, while there were periods of decline, the general trend was an increase in liver‐related mortality over the study period. These trends0020may reflect underlying shifts in public health policies, advances in medical treatments, and changing risk factors, including alcohol consumption, obesity, and viral hepatitis. The findings highlight the continued burden of liver diseases in the United States, particularly in high‐risk populations.

The observed increase in overall AAMR from 16.61 in 1999 to 18.93 in 2020 is indicative of an ongoing public health challenge. The earlier decline between 2002 and 2008 aligns with medical advances, particularly the introduction of direct‐acting antiviral therapies for hepatitis C [11, 12, 13]. However, the subsequent rise in AAMR after 2014 may be partially explained by the increasing prevalence of metabolic liver diseases, such as NAFLD, driven by rising obesity rates and other lifestyle factors [14, 15, 16]. This pattern mirrors global trends in the growing burden of metabolic diseases [17, 18].

Sex‐specific trends consistently showed higher mortality rates among men compared to women throughout the study period, aligning with known behavioral risk factors such as higher alcohol consumption and tobacco use in men [19]. The sharp increase in male mortality rates after 2014 suggests that while medical advancements initially reduced mortality, rising rates of alcohol abuse and metabolic disorders may have contributed to the reversal of these gains [20]. Although women also saw a gradual rise in mortality, particularly after 2008, the increase was less pronounced compared to men. This may reflect differences in the progression of liver disease and social factors affecting women's health, such as alcohol consumption and the growing impact of NAFLD [21].

Significant racial and ethnic disparities were observed throughout the study. Hispanic populations consistently had the highest AAMRs, a finding consistent with prior research indicating elevated liver disease mortality in this group [22, 23]. This disparity may be influenced by higher rates of obesity, diabetes, and genetic predispositions that contribute to this elevated risk [24, 25]. The fluctuations in mortality among Hispanic individuals, particularly the sharp increase post‐2008, may reflect broader socioeconomic factors such as access to care and economic disparities, as well as changing lifestyle patterns [26].

NH Black individuals also experienced fluctuating mortality rates, with a concerning increase in recent years. This trend could be attributed to factors such as limited access to care, delayed diagnoses, and high prevalence of comorbid conditions like hypertension and diabetes, which can exacerbate liver disease progression [27]. Conversely, NH Asian or Pacific Islander populations exhibited the lowest mortality rates throughout the study period, potentially due to protective dietary factors and more effective public health interventions [23]. However, a slight increase in mortality rates toward 2020 may be linked to changing risk factors, including alcohol consumption and metabolic syndrome [28].

A striking disparity was observed between urban and rural populations, with rural areas such as Micropolitan and Non‐Core (Nonmetro) regions exhibiting the highest AAMRs. These areas are likely to face greater challenges due to limited healthcare access, higher rates of alcohol consumption, and higher obesity rates [29]. The increasing AAMRs in these rural regions, particularly in Micropolitan areas, reflect the growing burden of liver disease in underserved populations, likely exacerbated by barriers to early detection and treatment [30]. In contrast, larger metro areas exhibited more stable trends, potentially reflecting better healthcare infrastructure, more timely diagnoses, and improved access to treatments [31].

At the regional level, the Southern United States reported the highest AAMR (18.9), which aligns with higher rates of obesity, alcohol consumption, and diabetes in this region [32]. The significant increase in mortality in the South toward 2020 underscores the critical need for targeted public health interventions that address these risk factors. States such as Texas, with the highest AAMR (25.7), could particularly benefit from efforts to improve healthcare access and implement preventive strategies to reduce liver disease‐related deaths [33]. Geographic trends also revealed varied mortality patterns across the Midwest and Northeast, where some regions showed a decline in mortality during certain periods, suggesting that region‐specific public health strategies and interventions may yield positive outcomes.

Future clinical implications of research on liver cirrhosis‐related mortality rates are significant, as they can guide the development of more effective prevention, diagnosis, and treatment strategies. By identifying key risk factors and mortality trends, clinicians can better stratify patients based on risk, allowing for earlier interventions and personalized care plans. Enhanced understanding of demographic and comorbidity patterns may also inform public health initiatives aimed at reducing the burden of cirrhosis through targeted education, lifestyle modification programs, and improved access to care. Ultimately, such research can contribute to evidence‐based policy changes and optimize resource allocation within healthcare systems to reduce mortality and improve outcomes for individuals living with liver cirrhosis.

## 5 Limitations

While this study provides valuable insights into liver disease mortality trends, several limitations must be considered. First, reliance on death certificate data introduces potential inaccuracies in coding and classification, which may lead to underreporting or misclassification of liver‐related deaths. This is particularly important in cases where liver disease is a contributing factor but not the underlying cause of death, leading to potential underestimation of disease burden. Prior research has shown that relying solely on ICD codes for cirrhosis may miss up to 47.2% of cases, while broader mention of liver disease on death certificates reduces this underdiagnosis to 19.4% (*p* < 0.0001), highlighting the variability and limitations of mortality data based on death certificates [34]. Additionally, the study did not account for specific risk factors such as alcohol use, obesity, hepatitis infection rates, or comorbidities like diabetes and hypertension, which would have helped further elucidate the underlying causes of the observed trends. Furthermore, the study did not capture the influence of advances in medical treatments for liver disease, such as hepatitis C therapies and NAFLD management, which may have impacted mortality rates, particularly during the early years of the study. Finally, although AAMRs were used to control for changes in age distribution, other key demographic shifts such as migration patterns, changes in population composition, and socioeconomic transitions were not explicitly accounted for. These unmeasured factors may have influenced the observed trends and represent an additional limitation of this descriptive analysis.

## 6 Conclusion

The findings of this study underscore the persistent public health challenge posed by liver‐related diseases in the United States. The increasing mortality rates, particularly among Hispanic populations, rural areas, and the Southern United States, highlight the need for targeted public health efforts. Although medical advancements have led to declines in mortality during some periods, the rising prevalence of obesity, alcohol consumption, and other social determinants of health has contributed to a subsequent increase in liver‐related deaths. These disparities call for policy interventions that expand access to preventive services, improve healthcare infrastructure in underserved regions, and promote culturally tailored education campaigns. Clinically, there is a need to integrate routine liver function screening in primary care settings, enhance early diagnosis of cirrhosis and metabolic liver disease, and ensure timely linkage to specialty care for at‐risk populations. Addressing these trends requires focused efforts on prevention, early diagnosis, and treatment, particularly for high‐risk populations.

## Conflicts of Interest

The authors declare no conflicts of interest.

## Supporting information


**Data S1:** Supporting Information.

## Data Availability

The data that supports the findings of this study are available in the S1 of this article.
