# Supplementary material for: Trends and Demographics of Liver Fibrosis and Cirrhosis‐Related Mortality Among Adults Living in the United States From 1999 to 2020: A CDC Wonder Analysis
Source: JGH Open. 2025 Sep 26;9(10):e70247. doi: 10.1002/jgh3.70247 (PMC12474855; doi:10.1002/jgh3.70247)
Supplement: Supplementary file 1 — Data S1: Supporting Information. [file JGH3-9-e70247-s001.docx]

**Table1: Annual Percentage Changes (APCs) in Liver Fibrosis and Cirrhosis-Related Mortality Trends in United States 1999-2020**

| **VARIABLE** | **YEAR INTERVAL** | **APC (95% CI)** | ***p*-value** |
| --- | --- | --- | --- |
| Overall  4 Joinpoints | 1999-2002 | 2.1672 | 0.288558 |
|  | 2002-2008 | -4.1562* | **0.000806** |
|  | 2008-2014 | 2.6520* | **0.009004** |
|  | 2014-2018 | -0.0870 | 0.956914 |
|  | 2018-2020 | 7.0284 | 0.057218 |
| Male  2 Joinpoints | 1999-2003 | 0.7538 | 0.624068 |
|  | 2003-2007 | -6.3601* | **0.016836** |
|  | 2007-2020 | 1.3408* | **0.000050** |
| Female  3 Joinpoints | 1999-2002 | 1.6754 | 0.376260 |
|  | 2002-2008 | -2.6955* | **0.005615** |
|  | 2008-2018 | 2.3619* | **0.000006** |
|  | 2018-2020 | 6.5198* | **0.040702** |
| Hispanic or Latino  1 Joinpoint | 1999-2008 | -4.0122* | **0.000013** |
|  | 2008-2020 | 0.8266* | **0.031421** |
| NH American Indian or Alaskan Native  1 Joinpoint | 1999-2010 | -4.2233* | **0.000056** |
|  | 2010-2020 | 3.7312* | **0.000116** |
| NH Asian or Pacific Islander  1 Joinpoint | 1999-2015 | -1.8161* | **0.000007** |
|  | 2015-2020 | 2.5073 | 0.067703 |
| NH Black or African American  4 Joinpoints | 1999-2003 | -0.6588 | 0.660105 |
|  | 2003-2008 | -5.9907* | **0.003321** |
|  | 2008-2014 | 2.0624 | 0.074473 |
|  | 2014-2018 | -2.9876 | 0.187781 |
|  | 2018-2020 | 5.7645 | 0.197630 |
| NH White  2 Joinpoints | 1999-2002 | 2.4420 | 0.363339 |
|  | 2002-2008 | -3.7225* | **0.005569** |
|  | 2008-2020 | 2.2874* | **0.000001** |
| Northeastern Region  4 Joinpoints | 1999-2002 | 0.8376 | 0.541181 |
|  | 2002-2008 | -4.5416* | **0.000064** |
|  | 2008-2015 | 1.1565* | **0.040487** |
|  | 2015-2018 | -2.3065 | 0.400807 |
|  | 2018-2020 | 6.7449* | **0.033714** |
| Midwestern Region  3 Joinpoints | 1999-2002 | 2.3462 | 0.237090 |
|  | 2002-2007 | -4.4747* | **0.002125** |
|  | 2007-2018 | 1.7610* | **0.000058** |
|  | 2018-2020 | 7.0106* | **0.049480** |
| Southern Region  2 Joinpoints | 1999-2003 | 2.2901 | 0.177394 |
|  | 2003-2007 | -4.8037 | 0.058187 |
|  | 2007-2020 | 2.0912* | **<0.000001** |
| Western Region  2 Joinpoints | 1999-2002 | 1.4825 | 0.664632 |
|  | 2002-2007 | -6.6072* | **0.005235** |
|  | 2007-2020 | 1.5078* | **0.000407** |
| Large Central Metro 4 Joinpoints | 1999-2002 | 0.4006 | 0.815106 |
|  | 2002-2008 | -5.2759* | **0.000111** |
|  | 2008-2014 | 1.8995* | **0.041890** |
|  | 2014-2018 | -1.8159 | 0.283586 |
|  | 2018-2020 | 6.1928 | 0.087105 |
| Large Fringe Metro  3 Joinpoints | 1999-2002 | 2.3961 | 0.270658 |
|  | 2002-2008 | -4.0287* | **0.000757** |
|  | 2008-2018 | 1.0323* | **0.013214** |
|  | 2018-2020 | 5.5049 | 0.140947 |
| Medium Metro  2 Joinpoints | 1999-2002 | 3.2257 | 0.290511 |
|  | 2002-2008 | -3.6829* | **0.010973** |
|  | 2008-2020 | 2.4313* | **0.000001** |
| Small Metro  2 Joinpoints | 1999-2003 | 1.8646 | 0.280235 |
|  | 2003-2008 | -3.4299* | **0.046386** |
|  | 2008-2020 | 2.5473* | **<0.000001** |
| Micropolitan (Nonmetro)  2 Joinpoints | 1999-2003 | 1.9844 | 0.213132 |
|  | 2003-2007 | -4.3211 | 0.077605 |
|  | 2007-2020 | 3.3885* | **<0.000001** |
| NonCore (Nonmetro)  2 Joinpoints | 1999-2002 | 3.8577 | 0.297466 |
|  | 2002-2008 | -2.2452 | 0.168631 |
|  | 2008-2020 | 3.9471* | **<0.000001** |

*Statistically significant *p-*values are highlighted bold

APC, annual percent change; CI, confidence interval

Table 2. Age-Adjusted Mortality Rates among Adults in the United States from 1999-2020 stratified by states.

| **STATES** | Age Adjusted Rate |
| --- | --- |
| Alabama | 19.2 |
| Alaska | 14.1 |
| Arizona | 16.5 |
| Arkansas | 16.5 |
| California | 16.6 |
| Colorado | 14 |
| Connecticut | 14.7 |
| Delaware | 16.5 |
| District of Columbia | 17.9 |
| Florida | 16.7 |
| Georgia | 14.8 |
| Hawaii | 13.6 |
| Idaho | 12 |
| Illinois | 14.9 |
| Indiana | 16.8 |
| Iowa | 10.8 |
| Kansas | 12.6 |
| Kentucky | 19.8 |
| Louisiana | 16.1 |
| Maine | 13.3 |
| Maryland | 15.2 |
| Massachusetts | 14.9 |
| Michigan | 16 |
| Minnesota | 12.1 |
| Mississippi | 18.3 |
| Missouri | 14.2 |
| Montana | 13.1 |
| Nebraska | 9.4* |
| Nevada | 15.9 |
| New Hampshire | 13.1 |
| New Jersey | 14.4 |
| New Mexico | 25.4** |
| New York | 12.6 |
| North Carolina | 16.9 |
| North Dakota | 10.8 |
| Ohio | 16.9 |
| Oklahoma | 19.9 |
| Oregon | 13.7 |
| Pennsylvania | 15.6 |
| Rhode Island | 19.7 |
| South Carolina | 18.6 |
| South Dakota | 11.3 |
| Tennessee | 20.8 |
| Texas | 25.7** |
| Utah | 12.3 |
| Vermont | 12.4 |
| Virginia | 15.5 |
| Washington | 13.9 |
| West Virginia | 22.2** |
| Wisconsin | 12 |
| Wyoming | 13.5 |

** denotes AAMRs of the States that fell in the top 90^th^ percentile

* denotes AAMRs of the States that fell in the lower 10^th^ percentile
